# X-ray imaging of chemically active valence electrons during a pericyclic reaction

**DOI:** 10.1038/ncomms6589

**Published:** 2014-11-26

**Authors:** Timm Bredtmann, Misha Ivanov, Gopal Dixit

**Affiliations:** 1 Max Born Institute, Max-Born-Strasse 2A, 12489 Berlin, Germany; 2 Blackett Laboratory, Imperial College London, London SW7 2AZ, UK

## Abstract

Time-resolved imaging of chemically active valence electron densities is a long-sought goal, as these electrons dictate the course of chemical reactions. However, X-ray scattering is always dominated by the core and inert valence electrons, making time-resolved X-ray imaging of chemically active valence electron densities extremely challenging. Here we demonstrate an effective and robust method, which emphasizes the information encoded in weakly scattered photons, to image chemically active valence electron densities. The degenerate Cope rearrangement of semibullvalene, a pericyclic reaction, is used as an example to visually illustrate our approach. Our work also provides experimental access to the long-standing problem of synchronous versus asynchronous bond formation and breaking during pericyclic reactions.

## Abstract (web summary)

X-ray scattering experiments give details of the electrons in a system, although typically this is dominated by core and inert valence electrons. Here, the authors report a method to follow changes in the chemically active valence electrons, and use it to study the reaction mechanism of a pericyclic reaction.

## Graphical abstract

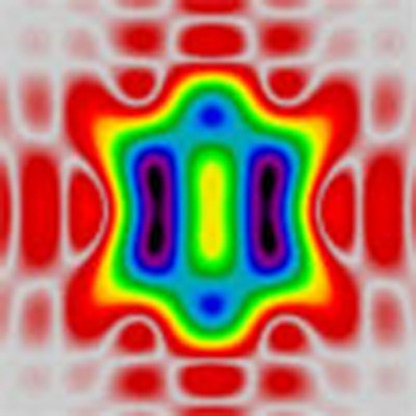

For over a century, X-ray and electron scattering have been indispensable in studying the structure of matter with atomic-scale spatial resolution. Thanks to enormous technological progress, it is now becoming possible to generate tunable, intense, ultrashort X-ray^1^,^2^,^3^ and electron pulses^4^,^5^, adding femtosecond temporal resolution to structural analysis. These pulses promise to provide time-resolved snapshots of physical, chemical and biological processes in individual molecules^6^,^7^,^8^. Atoms in molecules are glued together by valence electrons, which undergo ultrafast rearrangements during the formation and breaking of chemical bonds. Thus, the ability to follow the flow of valence electron density is paramount for ‘filming their motion’, and, ultimately, better understanding and controlling chemical reactions. The remarkable properties of modern ultrashort X-ray and electron pulses seem to offer a natural way for extending static scattering techniques into the domain of ultrafast electronic processes.

However, X-ray scattering from valence electrons is very weak in comparison with that from core electrons. The valence electron density contributes only a very small fraction to the total scattering pattern. Moreover, typically only a small fraction of the valence electron density participates actively in chemical reactions. These factors make use of X-ray scattering for time-resolved imaging of changes in chemically relevant parts of the valence electron density during complex chemical reactions extremely challenging. One way to circumvent the low sensitivity of X-ray scattering to valence electrons, proposed over four decades ago, is to use nonlinear X-ray scattering^9^. In a recent experiment, sum and difference frequency generation in the X-ray domain have been used to determine the valence electron density of diamond in real space, for a particular orientation^10^. However, realizing the time-resolved version of nonlinear X-ray scattering requires the triad of two optical and one ultrashort X-ray pulse with controlled variable time-delays between all three, by no means an easy task. Another option is to focus on the temporal behaviour of Bragg peaks. This approach has been successfully applied to imaging valence electron density in refs 6, 11, 12. However, it is limited to crystals, and requires subtracting of the total electron density at time zero from those at successive times, that is, the analysis of density differences.

Here we describe an effective and robust approach that allows us to extract changes of that part of the total electron density directly related to bond making and bond breaking during chemical reactions, that is, chemically active valence electron density, from the overall X-ray scattering pattern, which itself is dominated by the core electrons. This allows us to image the flow of valence electrons in space and time during a chemical reaction and thus solve one of the major problems, which has hampered the progress of time-resolved imaging of chemical reactions. Of course, many other experimental challenges remain, including better control of pump-probe jitter, better detectors, improved signal-to-noise ratios and so on. Our approach works in both the condensed and the gas phase, giving access to valence electron rearrangements in individual molecules. We illustrate our approach using the example of a very general pericyclic reaction—the degenerate Cope rearrangement of semibullvalene, sketched in Fig. 1. Our example also addresses another important and long-debated issue: is the breaking of old bonds during pericyclic reactions synchronous with the formation of new bonds? We show that our method distinguishes these two alternatives.

## Results

### The degenerate Cope rearrangement of semibullvalene

**Figure 1:**
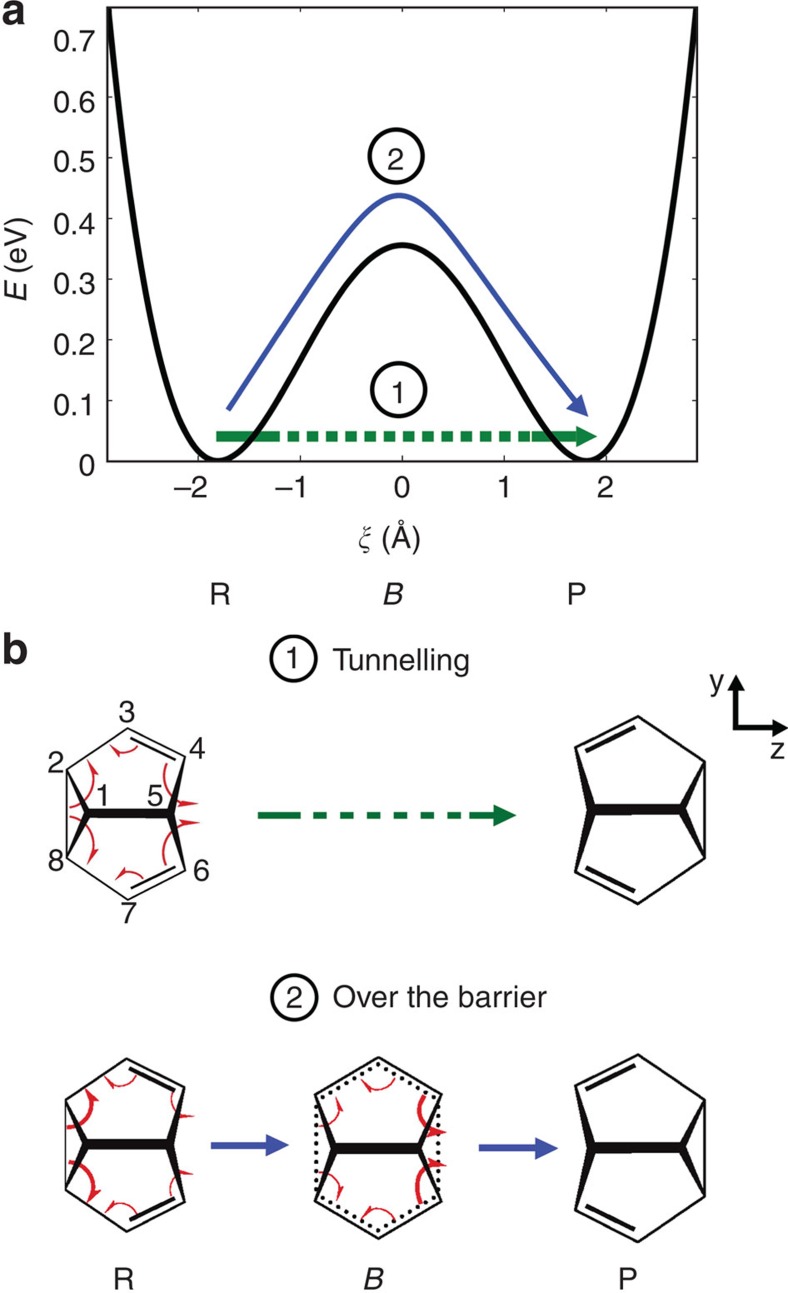
Degenerate Cope rearrangement of semibullvalene. (**a**) A cut through the potential energy surface along the effective reaction coordinate *ξ* from the reactant to the product including a sketch of the investigated reaction paths: tunnelling (green arrow), and over-the-barrier reaction (blue arrow). (**b**) Lewis structures of semibullvalene with the pincer-shaped arrows symbolizing electronic fluxes associated with chemical bond formation and breakage. The time sequences of these fluxes are sketched by the scale of the associated arrows (bonds associated with carbon atoms C4–C6 and C8–C2 are made and broken, respectively; double bonds change perpetually from C3=C4 and C6=C7 to C2=C3 and C7=C8). Synchronous bond formation and breakage is predicted for tunnelling, whereas, for the over-the-barrier reaction, asynchronous electronic rearrangement with dominant electronic fluxes out of the old bond from the reactant to the barrier, and reversed flux patterns from the barrier to the product are predicted.

Figure 1a shows effective reaction paths for the degenerate Cope rearrangement. In general, the reaction path strongly depends on the preparation of the reactants. Depending on the reactant energy, the Cope rearrangement can proceed by tunnelling (path 1) or over the barrier (path 2). Such alternative pathways are ubiquitous in many chemical reactions. They invariably trigger the question about synchronous versus asynchronous bond breaking and formation, debated over many decades for such pericyclic reactions^13^,^14^,^15^,^16^,^17^,^18^,^19^. So far, the debate has been purely theoretical.

Recent quantum mechanical *ab initio* calculations for the Cope rearrangement of semibullvalene in the electronic ground state predicted that the synchronicity of the underlying electronic bond-to-bond fluxes depends on the energy (temperature) of the reactant^16^,^17^. Synchronous bond formation and breaking was predicted for tunnelling, which is the dominant pathway at low energies (temperatures). In contrast, asynchronous bond breaking and formation was predicted for the over-the-barrier pathway, typical for high excitation energies (high temperatures). In this case, there should be a time-delay between the breaking of the old and the formation of the new bonds. Initiating pericyclic reactions at different excitation energies is possible using ultrashort optical pulses: down-chirped pump-dump and photo-impulsive methods have been proposed and experimentally demonstrated^20^,^21^.

### Imaging via time-resolved X-ray scattering

To simulate the time-resolved scattering pattern to image the Cope rearrangement, we have used the following expression for the differential scattering probability^22^,^23^,^24^


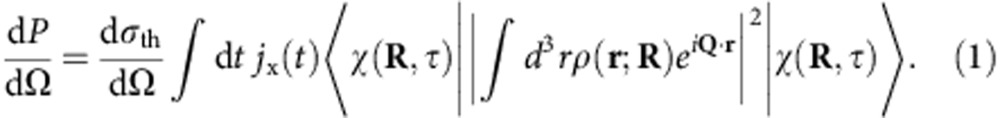


Here, d*σ*_th_/dΩ is the Thomson scattering cross-section, *j*_x_(*t*) is the fluence of the incident X-ray pulse at time *t*, *χ*(**R**, *τ*) corresponds to the nuclear wave packet with **R** denoting the set of nuclear coordinates with *τ* as the pump-probe delay-time, *ρ*(**r**; **R**) is the electronic density with **r** as electronic coordinate and **Q** is proportional to the momentum transfer of the scattered X-ray. *ρ*(**r**; **R**) is evaluated using quantum chemical calculations based on the density functional theory using the B3LYP functional with the cc-pVTZ basis sets by means of the MOLPRO program package^25^. The time-dependent nuclear Schrödinger equation is solved to compute the time evolution of *χ*(**R**, *τ*) along the reaction coordinate *ξ* describing the direct path from the reactant to the product as shown in Fig. 1a. Comparison with high-level *ab initio* methods shows that (i) the structures and energies along *ξ* are accurate in the present calculations and (ii) asynchronous bond making and breaking is a stable phenomenon, irrespective of the actual reaction path over the barrier^26^. The mean total energy along *ξ* for the reaction over the barrier is set to 1.25 eV, well above the potential barrier of 0.36 eV, and well below the first excited electronic state, which is about 4 eV higher in energy according to TDDFT calculation with the B3LYP functional using the 6-31G* basis sets^27^. Consequently, the dynamics along *ξ* is essentially decoupled from the other degrees of freedom^28^ and the reaction is well described within the Born–Oppenheimer approximation. For the calculation of the scattering patterns, an incident X-ray pulse with 20 keV photon energy and the detection of photons scattered up to 60° is assumed, yielding **Q**_max_=10 Å^−1^. The total scattering patterns encode the ground-state electron density. The excited electronic states and the electronic current density do not contribute to the total scattering pattern^22^,^23^,^24^,^29^,^30^.

### Distinction between images for different reaction paths

**Figure 2:**
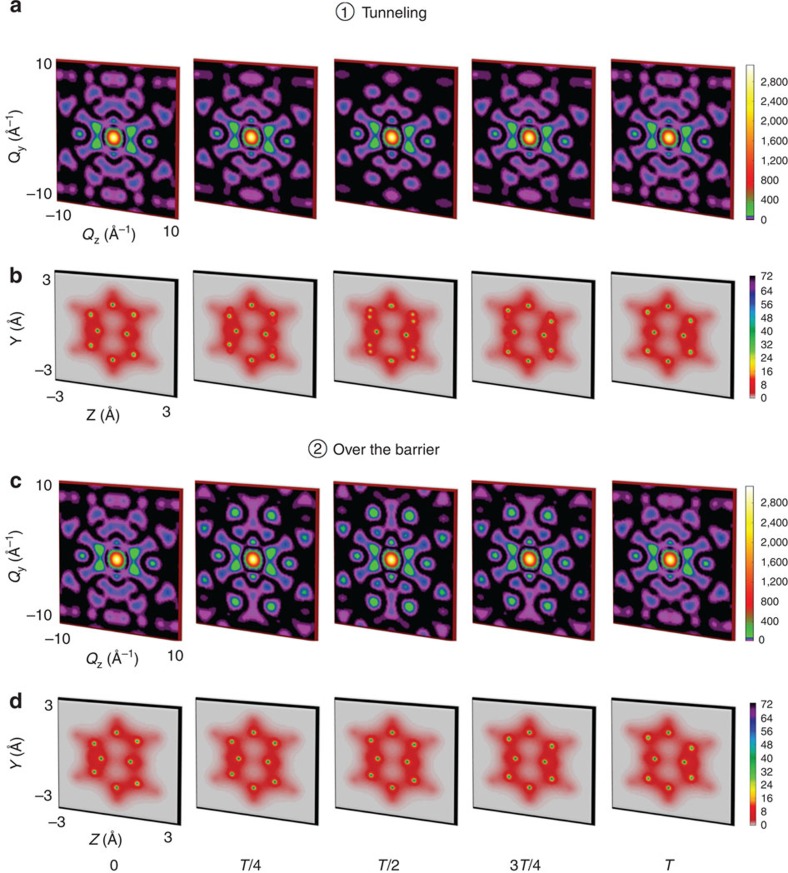
Time-resolved scattering patterns and corresponding total electron densities. (**a**) Scattering patterns, and (**b**) total electron densities during the Cope rearrangement of semibullvalene via tunnelling. (**c**) Scattering patterns, and (**d**) total electron densities for the reaction over the barrier. The time-resolved scattering patterns are in the *Q*_*y*_−*Q*_*z*_ plane (*Q*_*x*_=0) and the corresponding total electron densities are in the *y*−*z* plane (integrated along *x*-direction) at pump-probe delay times 0, *T*/4, *T*/2, 3*T*/4 and *T*. The time-resolved patterns are calculated until |**Q**_max_|=10 Å^−1^, which corresponds to an incident X-ray pulse with 20 keV photon energy and the detection of photons scattered up to 60°. The intensities of the patterns are shown in units of the differential scattering probability, d*P*_*e*_/dΩ, in both cases with 
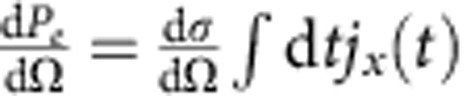
. The electron density is given in units of number of electrons per Å^2^.

Time-resolved scattering patterns in the *Q*_*y*_−*Q*_*z*_ plane (*Q*_*x*_=0) and the corresponding electron densities of space-fixed semibullvalene in the *y*–*z* plane as a function of the delay time at times *0*, *T/4*, *T/2*, *3T/4* and *T* are presented in Fig. 2. Here *T* is the reaction time for the Cope rearrangement of semibullvalene, ranging from *T*=24.2 fs (1 fs=10^−15^ s) for the over-the-barrier reaction to *T*=970 s for the reaction via tunnelling at cryogenic temperatures^31^. The static structure of semibullvalene in the gas phase has been measured using electron scattering^32^. Figure 2a,c shows that the time-resolved scattering patterns distinguish the over-the-barrier reaction from tunnelling. In the tunnelling case, the intensity of scattered photons, associated with high **Q**-values, diminishes from the reactant to the reaction intermediate at *T*/2 and then increases again between *T*/2 and *T* (see Fig. 2a). The opposite behaviour is observed for the over-the-barrier reaction. The difference between the two reaction paths is most prominent at *T*/2. The reader is referred to Supplementary Fig. 1 for further details of the total scattering patterns. The origin of these differences is in the different rearrangement dynamics of the carbon core electrons for the two paths, which dominate the total electron density as visually shown by localized yellow-green circles in Fig. 2b,d for tunnelling and over-the-barrier pathways, respectively. The contribution of the valence electrons is very diffuse.

**Figure 3:**
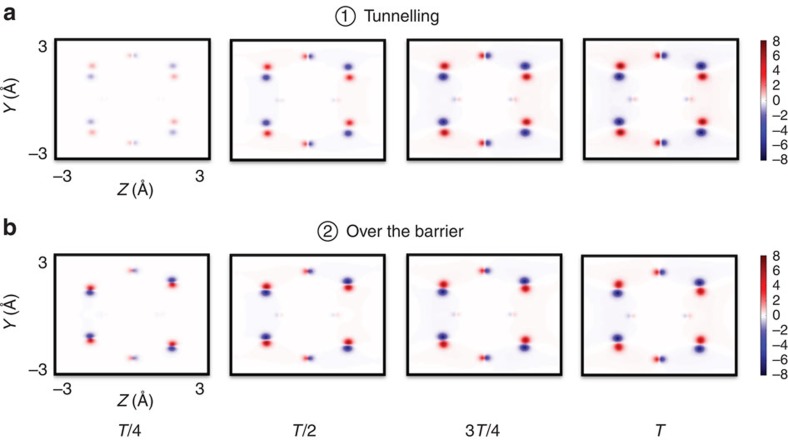
Density differences for the Cope rearrangement of semibullvalene. The total electron density at time zero is subtracted from the electron densities at later delay times during the course of the reaction via (**a**) tunnelling, and (**b**) for the over-the-barrier reaction. The density differences are presented in the *y*−*z* plane at pump-probe delay times *T*/4, *T*/2, 3*T*/4 and *T*.

Although the full time-resolved scattering patterns distinguish tunnelling from the over-the-barrier reaction, the changes in chemical bonding are hardly visible since the corresponding electron densities do not peak at the positions of the bonds^33^,^34^,^35^,^36^. Frequently one analyses total electron density differences to gain insight into valence electron rearrangements (see for example, refs 6, 11, 12). These are obtained by subtracting the total electron density at time zero from the successive snapshots of the total electron density. Such electron density differences in the *y*–*z* plane at times *T*/4, *T*/2, 3*T*/4 and *T* are shown in Fig. 3a,b for tunnelling and the over-the-barrier reaction, respectively. The contributions of core and inert valence electrons are much more pronounced in the electron density differences, hiding information from chemically active electrons. It is worth to mention that the analysis of the normalized density difference yields identical conclusions.

### Imaging chemically active electrons

In theoretical analysis of chemical bonding, topological analysis^33^,^34^,^35^,^36^ or a partitioning of the total electronic density based on localized molecular orbitals^37^,^38^ is typically used. In refs 16, 17, 18 such analyses enabled the prediction of synchronous versus asynchronous bond making and bond breaking, c.f. Fig. 1b. Specifically, the total electronic density was partitioned into a core electron density *ρ*_core_(**r**; **R**) (which accounts for the 16 carbon core electrons), a pericyclic electron density *ρ*_peri_(**r**; **R**) (which accounts for the six electrons associated with the mutation of the Lewis structure for the reactant into that of the product) and a density of the remaining valence electrons *ρ*_oval_(**r**; **R**), such that *ρ*(**r**; **R**)=*ρ*_core_(**r**; **R**)+*ρ*_peri_(**r**; **R**)+*ρ*_oval_(**r**; **R**). Although the pericyclic density has been established as a powerful theoretical tool for the analysis of pericyclic reactions, as well as to access the underlying bond-to-bond fluxes, no method exists to observe this quantity experimentally.

**Figure 4:**
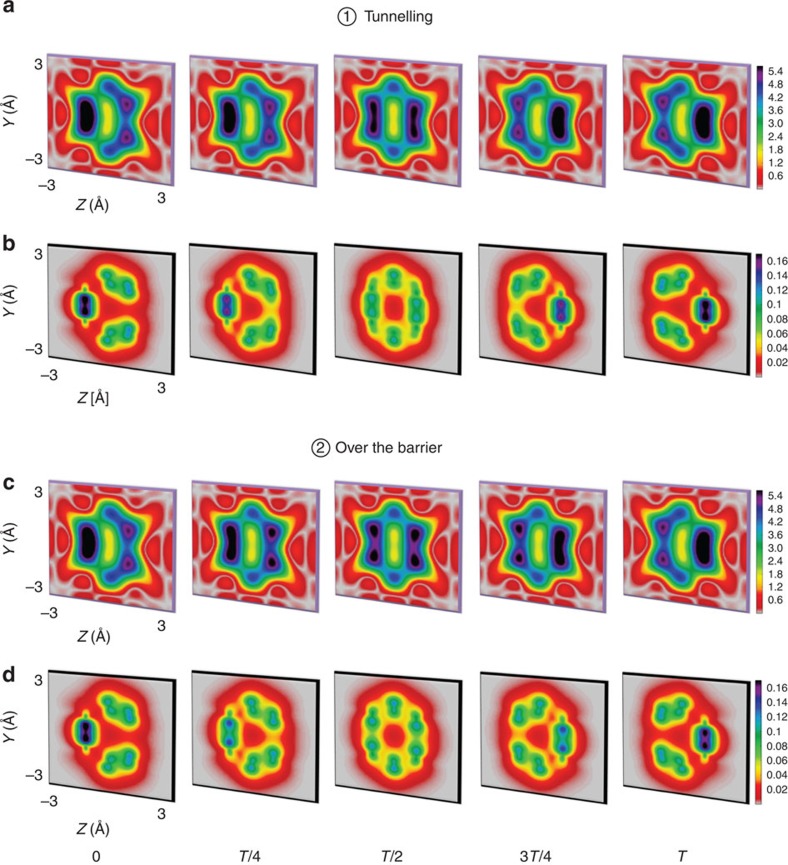
Reconstructed valence electron densities and the pericyclic electron densities. (**a**) Reconstructed valence electron densities obtained via the restricted **Q**-reconstruction method, and (**b**) the pericyclic electron densities (*ρ*_peri_(**r**; **R**)) obtained via partitioning the total electron densities for the Cope rearrangement of semibullvalene via tunnelling. (**c**) Reconstructed valence electron densities, and (**d**) *ρ*_peri_(**r**; **R**) for the over-the-barrier reaction. The electron densities are in the *y*−*z* plane at pump-probe delay times 0, *T*/4, *T*/2, 3*T*/4 and *T*. *ρ*_peri_(**r**; **R**) accounts for the six rearranging valence electrons according to the Lewis structures (cf. Fig. 1b). The reconstruction of the densities is performed using scattering intensity information up to |**Q**_limited_|=3.4 Å^−1^ from the full time-resolved scattering patterns as shown in Fig. 2. The electron density is given in units of number of electrons per Å^2^.

We now demonstrate how the chemically active valence electron densities, which carry invaluable information about chemical reactions and hence electronic bond-to-bond fluxes, can be directly accessed from the full scattering patterns: although there is no strict separation of the contributions from the core, inert valence and the chemically active valence electrons to the total scattering pattern, their relative contributions might be different in different regions of the **Q**-space. The delocalized valence electrons scatter weakly and should show their fingerprints mostly in the low **Q**-region of the scattering pattern. The well-localized core electrons, on the other hand, scatter strongly and contribute across the full scattering pattern. For the Cope rearrangement of semibullvalene, representing a whole range of pericyclic reactions, the chemically active valence electrons can indeed be brought to the fore to a remarkable extent by restricting the reconstruction to the relatively small momentum transfer, corresponding to the low **Q**-region, **Q**_max_>**Q**_limited_, as shown in Fig. 4. Performing this (restricted-Q) fourier transform from the Q-space back to the coordinate space requires knowledge of the phases associated with the full scattering pattern. Several phase-retrieval procedures exist to obtain the phase and reconstruct the total electron density from the scattering pattern for both crystalline and non-crystalline samples^39^,^40^. While in Fig. 4 the phase information is inferred from the theoretical calculations, we show in Supplementary Figs 2 and 3 that virtually identical results are obtained if the phase information is retrieved using a hybrid-input-output (HIO) algorithm, based on iterative fourier transformations, back and forth between the momentum space and real space^41^.

The retrieved electron densities in the *y*−*z* plane, using the restricted **Q**-region information, are presented in Fig. 4a,c for tunnelling and the over-the-barrier reaction, respectively. For comparison, the modelled pericyclic electron densities (*ρ*_peri_(**r**; **R**)) for tunnelling and the over-the-barrier reaction at times 0, *T*/4, *T*/2, 3*T*/4 and *T* are shown in Fig. 4b,d, respectively. A value of **Q**_limited_ equal to 3.4 Å^−1^ is used for the retrieval of electron densities using restricted-**Q** fourier transform, for Fig. 4a,c. To ensure robust reconstruction, we have varied **Q**_limited_, and find that the key features of the reconstructed densities are present for **Q**_limited_ ranging from 3 to 4.5 Å^−1^. For an illustration of the robustness of the results, the reader is referred to Supplementary Fig. 4.

Figure 4 shows that the hitherto dominant contribution from the core and inert valence electrons has been considerably diminished, while the contribution of the hitherto hidden chemically active electrons has been considerably increased. We observe that the reconstructed electron densities have more strength than *ρ*_peri_(**r**; **R**), which indicate that the reconstructed electron densities contain the contributions from pericyclic electrons as well as from other valence and core electrons, whereas *ρ*_peri_(**r**; **R**) contains the contributions from pericyclic electrons alone. However, the reconstructed electron densities are in excellent agreement with *ρ*_peri_(**r**; **R**), which provides direct insight into the reaction mechanism. We note that the restricted **Q**-reconstruction method is analogous to fourier filtering techniques of image analysis with a low-pass square-shaped filter. In particular, the ‘ringing’ in the retrieved electron densities is related to the sharp edge of the square filter. Typically, a low-pass filter blurs the image. Here, however, this blurring is beneficial: we see that it brings out the chemically active electron density. Regarding the applicability of our method, the key requirement is the ability to measure wide-angle scattering pattern up to **Q**_max_, which enables atomic-scale spatial resolution and faithful reconstruction of the lost phase of the scattering pattern using a phase-retrieval algorithm.

## Discussion

The issue of synchronous versus asynchronous bond formation and breakage can indeed be addressed using the reconstructed electron densities. The reconstructed electron densities for the reaction intermediate at *T*/2 show that in the tunnelling case, the reaction is synchronous, whereas it is indeed asynchronous in the over-the-barrier case. At *T*/2, the electronic flux out of the old bond, centred around *z*=−1 Å and *y*=0 Å, is larger for the over-the-barrier reaction than for tunnelling (disconnected and connected black contours in the vicinity of the old bond, respectively). At the same time, the flux into the new bond, centred around *z*=1 Å and *y*=0 Å, is smaller for the over-the-barrier reaction as compared with the tunnelling scenario.

In conclusion, using the degenerate Cope rearrangement of semibullvalene as an example, we have demonstrated a powerful approach to retrieve valence electron density from the full time-resolved scattering pattern using the restricted **Q**-reconstruction method. The phase of the scattering pattern needed for the reconstruction can be obtained from the full, unrestricted-**Q**, scattering pattern. Our approach enables us to image the instants of bond formation and breakage during chemical processes and resolve experimentally the long-standing debate of synchronous versus asynchronous bond formation and breakage during chemical reactions. Of course, our approach also applies for imaging chemically active valence electron densities in static structures and non-adiabatic chemical reactions^42^. It is general and applicable not only to ultrafast time-resolved X-ray scattering but also to electron scattering, which are emerging as the methods of choice for imaging ultrafast chemical and biological processes with atomic-scale spatial and temporal resolutions. The feasibility of single-molecule imaging in the gas phase using X-ray^43^ and electron^44^ scattering has recently been experimentally demonstrated, which provides the first frame of time-resolved chemical processes. Three-dimensional molecular alignment is also experimentally feasible^45^, but combining such alignment with X-ray imaging is a major challenge. The problem may be circumvented by using Van der Waals crystals, which will also greatly enhance the required signal.

The notion of quantum fluxes along with their respective densities played a pivotal role for gaining insight about complex chemical reactions. However, the concept of the fluxes and their densities has so far been limited to theoretical modelling. Our reconstruction allows us to visualize (probe) the fluxes and their densities. The imaged electron density can be directly used to determine bond-to-bond electronic fluxes during chemical reactions, essential for the deeper understanding of complex chemical reactions. Their direct imaging opens new possibilities for manipulating and controlling chemical processes.

## Methods

### Theoretical modelling

We describe the coupled electronic and nuclear motion during the Cope rearrangement of semibullvalene in the electronic ground state quantum mechanically within the framework of the Born–Oppenheimer approximation. This is justified by the large energy gap of at least 4 eV to the first excited electronic state according to TDDFT calculations using the B3LYP functional with the 6-31G* basis sets^27^, confirmed by multi-reference configuration interaction calculations with the cc-pVTZ basis sets. Consequently, the molecular wavefunction is approximated as


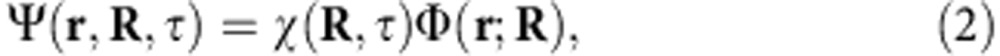


where **r** and **R** denote the spatial coordinates of the *N* electrons and *M* nuclei, respectively. The corresponding time-dependent electronic density is given by


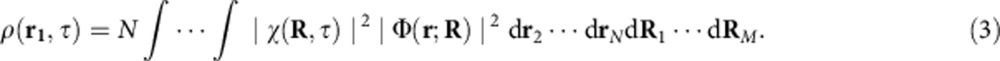


According to transition state theory calculations with quantum corrections, the Cope rearrangement of semibullvalene proceeds for temperatures below 40 K by coherent tunnelling involving the lowest doublet of nuclear eigenstates within *T*=970 s^31^. Hence, the nuclear density |*χ*(**R**,*τ*)|^2^ for tunnelling consists essentially of two parts representing the reactant and the product, centred at **R**_R_ and **R**_P_, respectively, superimposed according to


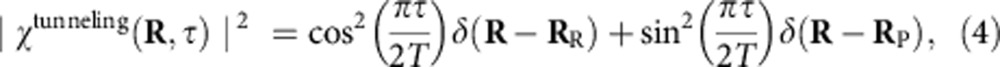


where *δ* denotes the Dirac distribution^17^. Inserting equation (4) into equation (3) and performing the integration yields the time-dependent electron density for the tunnelling scenario. For the reaction over the barrier, the time-dependent nuclear Schrödinger equation is solved to compute the time evolution of *χ*^otb^(**R**, *τ*) along the reaction coordinate *ξ*, describing the direct path from the reactant to the product corresponding to synchronous nuclear motion. The underlying potential energy surface and the corresponding electron densities are evaluated at the B3LYP/cc-pVTZ level of theory employing the Molpro program package^25^, yielding excellent agreement with high-level multi-reference *ab initio* calculations^26^. The mean total energy along *ξ* is set to 1.25 eV, well above the potential barrier of 0.36 eV. Consequently, the dynamics along *ξ* is essentially decoupled from the other degrees of freedom^28^. It shall be added that asynchronous bond making and bond breaking in the reaction over the barrier is a stable phenomenon in the investigated pericyclic reaction, which is even more pronounced when deviating from the direct path as shown in refs 18, 26.

### Retrieval of chemically active valence electron densities

The full time-resolved scattering pattern provides only the scattering amplitude while the phase is lost in the measured scattering signal. There exists a variety of phase-retrieval procedures to reconstruct the phase from the scattering amplitude and therefore reconstruct the total electron density of crystalline and non-crystalline samples^39^,^40^. In the present case, we have used the HIO algorithm, based on iterative fourier transformations, back and forth between the momentum space and real space^41^. Combining the retrieved phase and the scattering amplitude, the total electron densities for the Cope rearrangement of semibullvalene can be reconstructed in the present case by performing two-dimensional fourier transformation over the extended Q-range equal to **Q**_max_=10 Å^−1^. For the reconstruction of the chemically active electron density, the two-dimensional fourier transformation for the restricted range of the photon-momentum transfer, that is, |**Q**_limited_|=3.4 Å^−1^ is performed. The reader is referred to Supplementary Figs 2 and 3 for a detailed discussion of our implementation of the HIO algorithm.

To ensure the robustness of our approach for the retrieval of chemically active valence electron densities, the range of |**Q**_limited_| is varied from 3 to 4.5 Å^−1^, and the key features of the retrieved electron densities are present within this range of |**Q**_limited_|. The reader is referred to Supplementary Fig. 4 for the corresponding illustrations.

## Author contributions

G.D. conceived the idea of the project. T.B. performed the calculations. T.B., M.I. and G.D. analysed the results and wrote the manuscript.

## Additional information

**How to cite this article:** Bredtmann, T. *et al*. X-ray imaging of chemically active valence electrons during a pericyclic reaction. *Nat. Commun.* 5:5589 doi: 10.1038/ncomms6589 (2014).

## Supplementary Material

Supplementary Information Supplementary Figures 1-4, Supplementary Methods and Supplementary References
