# Supplementary material for: X-ray imaging of chemically active valence electrons during a pericyclic reaction
Source: Nat Commun. 2014 Nov 26;5:5589. doi: 10.1038/ncomms6589 (PMC4263170; doi:10.1038/ncomms6589)
Supplement: Supplementary Information — Supplementary Figures 1-4, Supplementary Methods and Supplementary References [file ncomms6589-s1.pdf]

Supplementary Information

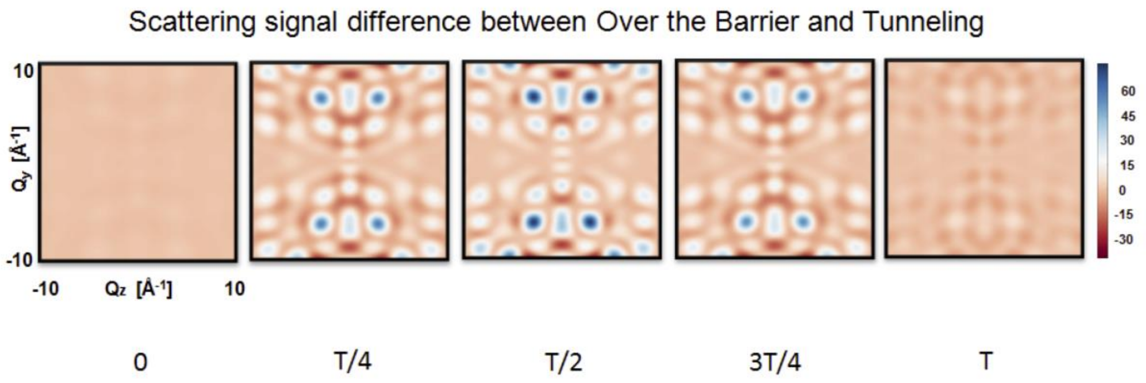

Supplementary Figure 1. Difference scattering patterns in the $Q_y - Q_z$ plane (Q_x = 0) for the Cope rearrangement of semibullvalene at pump-probe delay times 0, T/4, T/2, 3T/4 and T. Here, the scattering signal corresponding to tunneling is subtracted from the scattering signal corresponding to the over the barrier reaction.

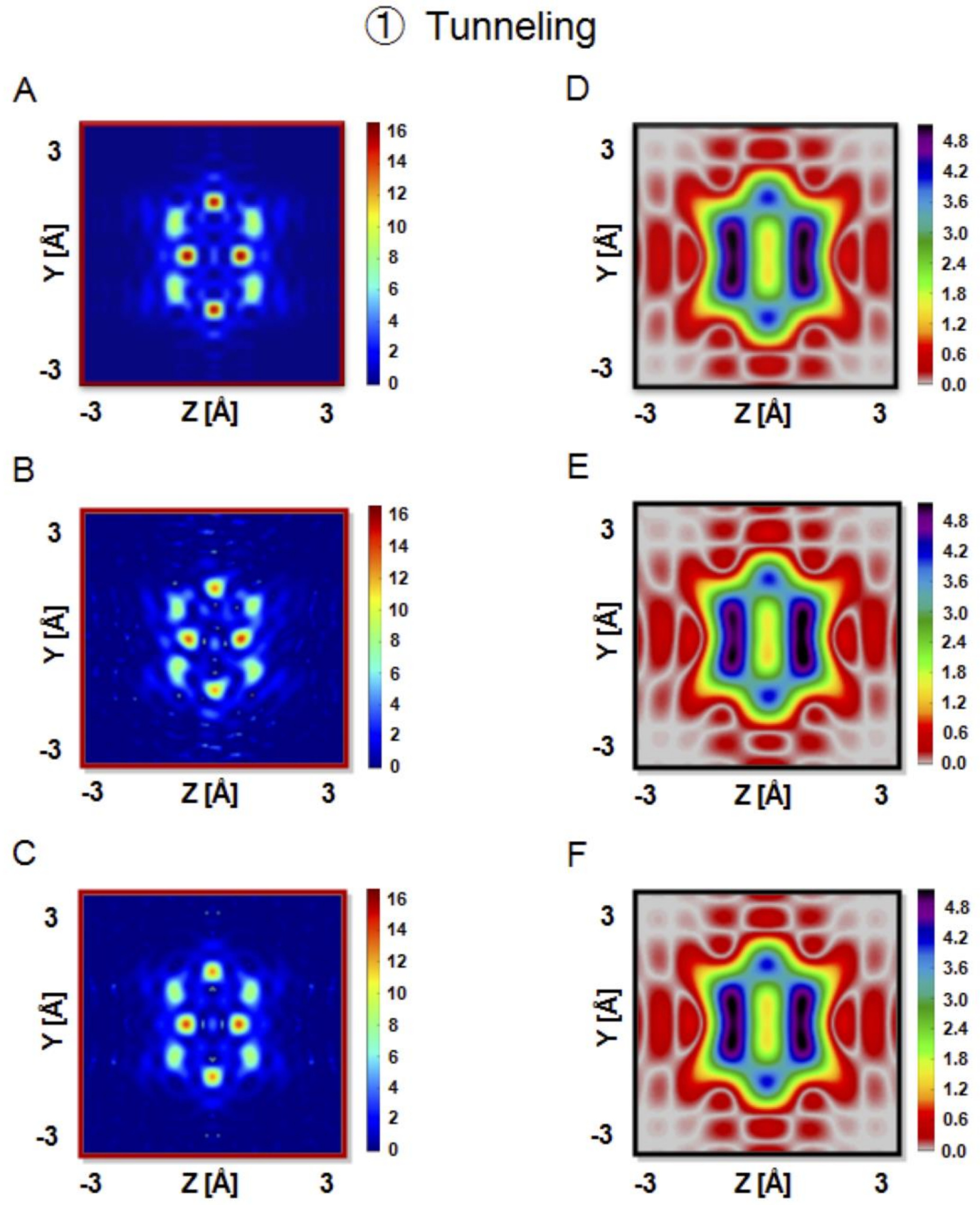

Supplementary Figure 2. Total electron densities (left) and chemically active electron densities (right) for the Cope rearrangement of semibullvalene in the tunneling regime, at T/2. The densities are reconstructed using the exact phase information from the theoretical calculations (A and D), using the retrieved phase information by means of the Hybrid-Input-Output (HIO) algorithm after 100 iteration (B and E) and using further 100 iterations of the HIO algorithm while imposing additional molecular symmetry constraint (C and F). Values of $\mathbf{Q}_{max}$ = 10 $Å^{-1}$ and $\mathbf{Q}_{limited}$ = 3.4 $Å^{-1}$ are used.

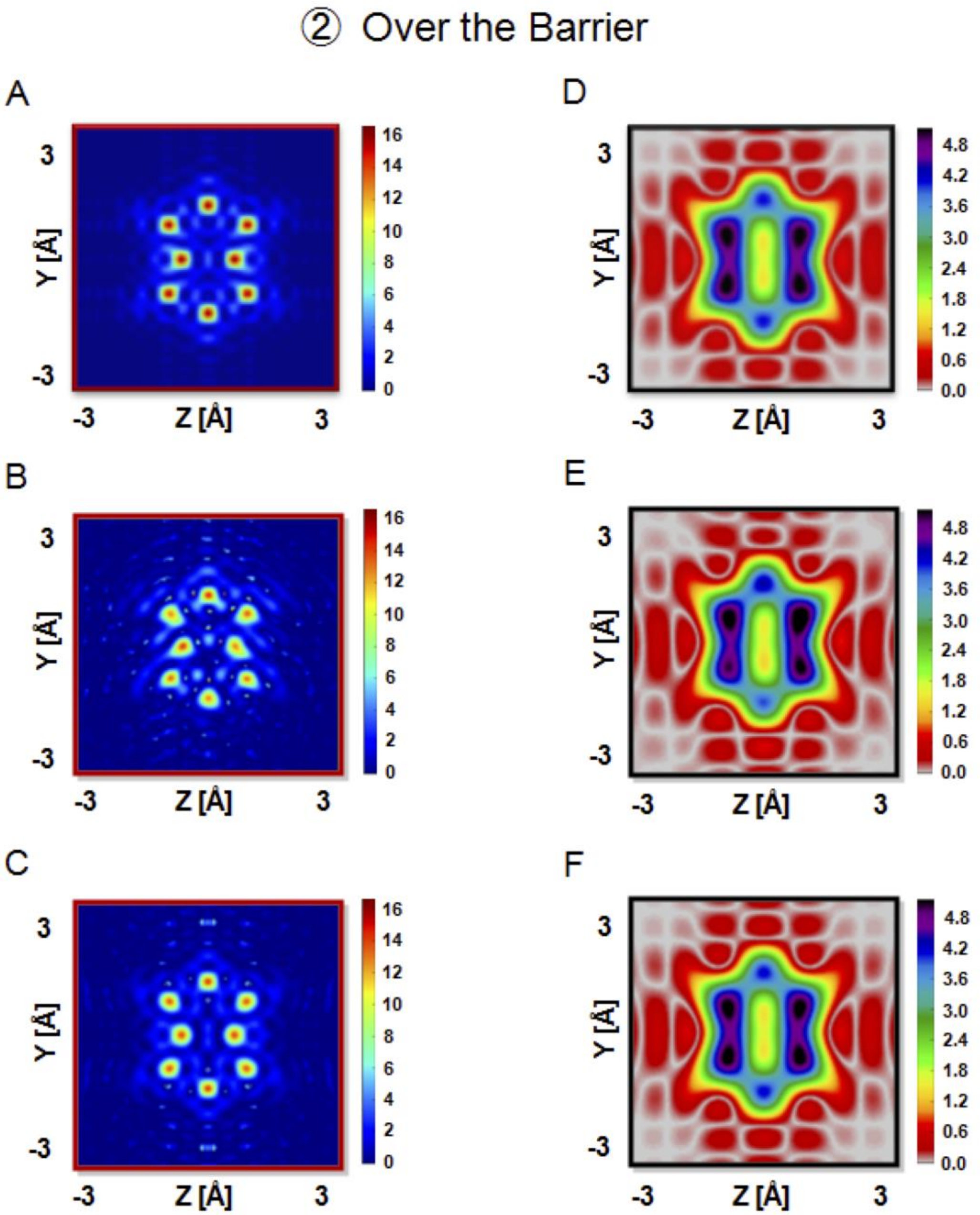

Supplementary Figure 3. Same as Supplementary Figure 2 for the Cope rearrangement of semibullvalene proceeding over the barrier.

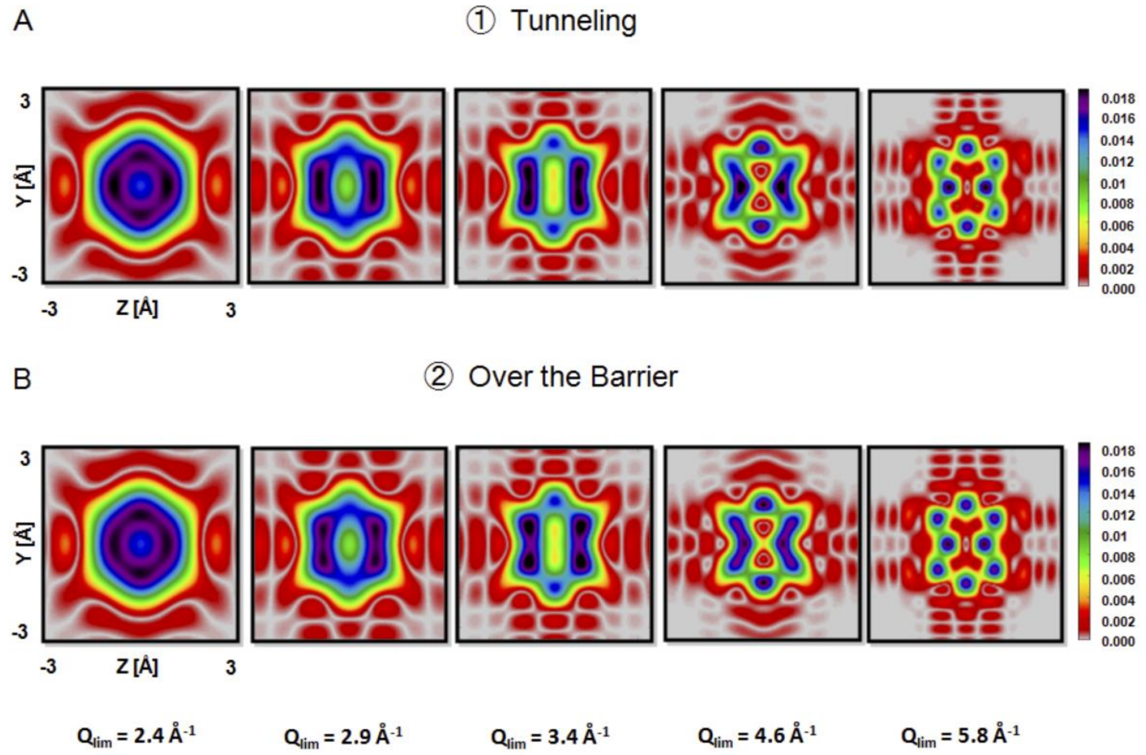

Supplementary Figure 4. The retrieved chemically active electron density at reaction time T/2 for (A) tunneling and (B) the over the barrier reaction as a function of $\mathbf{Q}_{\text{limited}}$ ranging from 2.4 Å^{-1} to 5.8 Å^{-1}.

Supplementary Methods

Here, we present further details for the imaging of chemically active electron density during a pericyclic reaction, the degenerate Cope rearrangement of semibullvalene, using time-resolved x-ray scattering. At first, the differences in the total scattering patterns for the reaction via tunneling and over the potential barrier are discussed. Subsequently, it is shown that the phase associated with the scattering pattern may be retrieved from the knowledge of the scattering amplitude alone by means of the Hybrid-Input-Output algorithm, allowing the faithful reconstruction of both the total electron density and the chemically active electron density. Finally, the robustness of the proposed method for imaging the chemically active electron density is demonstrated.

DETAILS OF TOTAL TIME-RESOLVED SCATTERING PATTERNS

Supplementary Figure 1 quantifies the differences in the time-resolved scattering patterns associated with the Cope rearrangement of semibullvalene in the tunneling and the over the barrier regimes. Supplementary Figure 1 shows the corresponding differences in the total scattering patterns, obtained by subtracting the scattering signal corresponding to tunneling from the signal corresponding to the over the barrier reaction. The differences are more pronounced at larger $\mathbf{Q}$, which provide another indication that the scattering patterns as well as the total electron densities are dominated by the core and inert valence electrons. Also, the difference scattering pattern at time T is not identical to zero. The reason for this non-zero difference scattering pattern is attributed to the dispersion of the nuclear wave packet in the over the barrier reaction, which is absent for the reaction occurring via tunneling [1].

PHASE RETRIEVAL FROM THE TOTAL SCATTERING PATTERN

In the simulations presented in the main part of the article, both the scattering amplitude, $|F(\mathbf{Q},t)|$, and the associated phase, $\Phi(\mathbf{Q},t)$, are obtained by Fourier transforming the time-dependent electron density $\rho(\mathbf{r},t)$, see the main text for further details. In experiments, however, the full time-resolved scattering pattern provides only the

scattering amplitude while the phase is lost during the scattering process. In the following, we show that our method works when only $|F(\mathbf{Q}, t)|$ is known. We employ the Hybrid-Input-Output (HIO) algorithm [2, 3] to reconstruct the phase $\Phi(\mathbf{Q}, t)$. The chemically active electron density is then obtained by performing the restricted-**Q** inverse Fourier transformation of $|F(\mathbf{Q}, t)|$ combined with the reconstructed phase information.

In the iterative HIO four-step algorithm, the *n*-th iteration is given as follows:

First step: Fourier transform the total electron density, $\rho_n(\mathbf{r})$ yielding $F_n(\mathbf{Q})$.

Second step: Calculate $F_n'(\mathbf{Q}) = |F(\mathbf{Q})| \times e^{i\Phi_n(\mathbf{Q})}$, where $\Phi_n(\mathbf{Q})$ is the phase associated with $F_n(\mathbf{Q})$ from **First step** and $|F(\mathbf{Q})|$ is the modulus of the calculated scattering pattern (Figures 2A and 2C in the main text) corresponding to the 'measured' scattering pattern (Fourier Domain Operation).

Third step: Inverse Fourier transform $F_n'(\mathbf{Q})$ to give $\rho_n'(\mathbf{r})$.

Fourth step: Apply the object domain operation

$$\rho_{n+1}(\mathbf{r}) = \begin{cases} \rho_n'(\mathbf{r}), & \text{for } \mathbf{r} \in S \text{ and } \rho_n'(\mathbf{r}) \geq 0 \\ \rho_n(\mathbf{r}) - \beta\rho_n'(\mathbf{r}), & \text{else} \end{cases}$$

to obtain the new estimate of the total electron density, $\rho_{n+1}(\mathbf{r})$.

In our calculations, the parameter β is set to 0.9 [4], and the pre-defined support S comprises -9.7 Å $\leq Y \leq 9.7$ Å and -9.7 Å $\leq Z \leq 9.7$ Å. The initial estimate of the total electron density is obtained by setting the phase associated with $|F(\mathbf{Q})|$ equal to zero. The signal $|F(\mathbf{Q})|$ is calculated until $\mathbf{Q}_{max} = 10$ Å^{-1}, c.f. Figure 2A and 2C. For $|\mathbf{Q}| > |\mathbf{Q}_{max}|$, $|F(\mathbf{Q}, t)|$ is padded with zeros to obtain spatial resolution of $\Delta Y = \Delta Z = 0.2$ Å for the reconstructed electron densities.

The resulting reconstructed total electron densities at T/2, after 100 iterations, are shown for the tunneling and the over the barrier reaction in Supplementary Figures 2B and 3B, respectively. The corresponding chemically active electron densities are shown in

Supplementary Figures 2E and 3E. For the restricted-**Q** transformation, we have used $\mathbf{Q}_{\text{limited}}$ = 3.4 Å^{-1}, c.f. middle panels in Figures 4A and 4C. For comparison, the corresponding reconstructed densities using the exact phase information are shown in Supplementary Figures 2A and 2D for the tunneling scenario and in Supplementary Figures 3A and 3D for the reaction over the barrier. Good agreement is obtained between the total and chemically active electron densities obtained using the exact phase and the phase obtained via the HIO algorithm. Most importantly, clear differences in the bond making and bond breaking in the two reaction paths are visible from the chemically active electron densities. Furthermore, it is worth noting that for the tunneling reaction, the core electrons associated with bond making and bond breaking (atoms 2, 4, 6 and 8, c.f. Figure 1B) are smeared out, even when the exact phase information is used (Supplementary Figure 2A). This effect occurs since the densities presented here are reconstructed from the total scattering pattern limited to $\mathbf{Q}_{\text{max}}$ = 10 Å^{-1}. On the other hand, the total densities directly obtained from the Quantum Chemistry calculation show clear separation between these core electrons, c.f. Figure 2B.

The results can still be improved by exploiting the symmetry of the given chemical re-action, which can be inferred from the reconstructed total electron densities. For example, adding the C_{2v}-symmetry at time T/2 for the Cope rearrangement of semibullvalene as additional object domain operation in the last step of the HIO algorithm leads to significant improvement. Performing 100 additional iterations yields excellent agreement with the densities that use exact phase information $\Phi(\mathbf{Q}, T/2)$, see Supplementary Figures 2C and 2F for the tunneling and Supplementary Figures 3C and 3F for the over the barrier reaction.

ROBUSTNESS OF THE RESTRICTED Q-FOURIER TRANSFORM METHOD

To verify the robustness of the restricted **Q**-Fourier transform method, Supplementary Figure 4 shows a series of reconstructed electron densities both for the tunneling and the over the barrier reaction at half time, T/2, as a function of $\mathbf{Q}_{\text{limited}}$. Using scattering information in the range 3 Å^{-1} < $\mathbf{Q}_{\text{limited}}$ < 4.5 Å^{-1}, clear distinctions in the formation

and breaking of the chemical bonds can be observed: In particular, less electron density is observed in the regions of the new and the old bond for the over the barrier reaction as compared to synchronous bond making and bond breaking in the case of tunneling, resulting in asynchronous bond making and bond breaking in the over the barrier reaction. On the other hand, for values of $\mathbf{Q}_{\text{limited}} < 3$ Å^{-1}, these processes are blurred while for values of $\mathbf{Q}_{\text{limited}} > 4.5$ Å^{-1}, the core electrons start dominating the reconstructed densities.

Supplementary References:

[1] Bredtmann, T. & Manz, J. Electronic Bond-to-Bond Fluxes in Pericyclic Reactions: Synchronous or Asynchronous? *Angewandte Chemie International Edition* **50**, 12652–12654 (2011).

[2] Fienup, J. R. Phase retrieval algorithms: a comparison. *Appl. Opt.* **21**, 2758–2769 (1982).

[3] Fienup, J. R. Reconstruction of a complex-valued object from the modulus of its Fourier trans- form using a support constraint. *J. Opt. Soc. Am. A* **4,** 118–123 (1987).

[4] Marchesini, S. *et al.* X-ray image reconstruction from a diffraction pattern alone. *Physical Review B* **68**, 140101 (2003).
